# Imaging myelin degradation in ex vivo prefrontal cortex tissue blocks in Alzheimer's disease and chronic traumatic encephalopathy

**DOI:** 10.1002/alz.70582

**Published:** 2025-08-22

**Authors:** Anna Novoseltseva, Gulce Kureli, Shuaibin Chang, Jiarui Yang, Precious Dominique Antinew, Arjun Chandra, Samer Berghol, Elizabeth Spurlock, Ann C. McKee, Bertrand Huber, Hui Wang, David A. Boas, Irving J. Bigio

**Affiliations:** ^1^ Department of Biomedical Engineering Boston University Boston Massachusetts USA; ^2^ Department of Electrical & Computer Engineering Boston University Boston Massachusetts USA; ^3^ Division of Graduate Medical Sciences Chobanian & Avedisian School of Medicine Boston University Boston Massachusetts USA; ^4^ Department of Computer Science Boston University Boston Massachusetts USA; ^5^ Department of Neurology Boston University Alzheimer's Disease Research Center Boston University CTE Center Boston University School of Medicine Boston Massachusetts USA; ^6^ Department of Radiology, Athinoula A. Martinos Center for Biomedical Imaging Department of Radiology Massachusetts General Hospital/Harvard Medical School Charlestown Massachusetts USA

**Keywords:** Alzheimer's disease, birefringence microscopy (BRM), chronic traumatic encephalopathy, gray matter myelin defects, label‐free imaging techniques, myelin degeneration, polarization‐sensitive optical coherence tomography (PS‐OCT)

## Abstract

**INTRODUCTION:**

Alzheimer's disease (AD) and chronic traumatic encephalopathy (CTE) are tauopathies with gray matter (GM) myelin changes that are challenging to assess with standard imaging. New methods are needed to quantify myelin integrity in autopsy brain tissues.

**METHODS:**

We used polarization‐sensitive optical coherence tomography (PS‐OCT) to measure bulk tissue relative retardance and birefringence microscopy for high‐resolution imaging of myelin degradation. Samples included five AD, five CTE, and four age‐matched normal controls.

**RESULTS:**

When controlling for age and postmortem interval, no statistically significant differences in white matter retardance or GM myelin defect density were observed between groups. The age difference between controls (64 ± 4.7 years, mean ± SD) and disease groups (80.3 ± 7 years) emerged as an important confounding factor. Amyloid beta and tau staining showed weak correlations with myelin defects.

**DISCUSSION:**

Our label‐free approach enables large‐volume imaging of brain tissue, a valuable tool for studying myelin changes in neurodegenerative diseases.

**Highlights:**

- Multi‐modal assessment of myelin integrity using polarization‐sensitive optical coherence tomography (PS‐OCT) and high‐resolution birefringence microscopy.
- Age emerged as a critical confounding factor; no significant disease differences were found.
- Weak correlation between myelin defects and deposition of amyloid beta/tau was found in prefrontal gray matter.
- Label‐free optical methods enable high‐resolution, large‐volume imaging of myelin.

## 1 BACKGROUND

Alzheimer's disease (AD) and chronic traumatic encephalopathy (CTE) are progressive dementias characterized by accumulations of abnormally phosphorylated tau protein manifesting clinically.^1^ AD is a leading cause of death among the elderly, and it is most often sporadic.^2^ CTE is causally linked to repetitive head impacts, such as those sustained during contact sports participation and military service.^3^, ^4^, ^5^, ^6^ AD is neuropathologically defined by the presence of neuritic plaques containing amyloid beta (Aβ) protein and neurofibrillary tangles (NFTs).^7^ CTE is defined by the irregular perivascular accumulation of hyperphosphorylated tau (p‐tau) in neurons, with a predilection for the depths of the sulci of the cerebral cortex.

Beyond tau and Aβ pathology, it is well established in both AD and CTE research that axonal damage plays a critical role in disease progression,^8^ often contributing to cognitive decline and executive dysfunction.^9^, ^10^ In AD, brains exhibit dystrophic axons and synaptic loss,^11^, ^12^ which are well‐documented markers of neurodegeneration. Diffusion tensor imaging (DTI) studies have shown alterations in white matter (WM) structures that are correlated with axonal degeneration and synaptic loss.^9^, ^10^ Accumulation of tau is implicated in this degeneration, disrupting phosphotransferase activities, calcium homeostasis, and axonal transport, ultimately contributing to NFT formation and axonal degeneration.^13^ Similarly, post‐mortem analysis of CTE cases demonstrates widespread diffuse axonal injuries and axonal swellings.^10^ In both conditions, DTI studies reveal a strong correlation between axonal atrophy and cognitive decline.^14^, ^15^, ^16^, ^17^ For instance, in CTE, studies have shown a link between the number of years of contact sports played and WM rarefaction,^18^ with a reduction in the number of oligodendrocytes further emphasizing potential myelin damage.^19^ Moreover, axonal injury has been proposed as a potential trigger for p‐tau pathology in CTE.^20^ The importance of discussing axonal damage lies in its frequent correlation with myelin damage, often via mechanisms like Wallerian degeneration, where axonal injury leads to secondary myelin degradation,^21^, ^22^ further amplifying neurodegenerative processes in both AD and CTE.

In AD, Aβ plaques are found predominantly in poorly myelinated or demyelinated regions of both gray matter (GM) and WM.^23^ Although most literature on myelin changes in AD and CTE focuses on WM, the GM merits particular attention, as it represents the primary site of early neuropathological changes, including initial tau deposition and synaptic loss.^8^, ^24^ Recent studies have implicated a connection between impaired cholesterol clearance and limited remyelination in AD mouse models.^23^ Aβ toxicity causes microtubule depolymerization and neuritic beading, resulting in axonal trafficking failure.^23^ Although WM rarefaction and myelin degradation are commonly observed in AD and CTE, traditional histological methods are limited to thin sections with small volumes and are compromised by distortion from cutting, slice mounting, dehydration, and staining.^25^, ^26^, ^27^

We seek to determine the potential for unlabeled optical imaging to assess myelin integrity, both over large volumes at mesoscopic scale and also at high resolution, single‐axon scale. Polarization‐sensitive optical coherence tomography (PS‐OCT), with blockface imaging of the WM incorporating serial‐sectioning,^28^ is capable of reconstructing large‐scale volumetric structures,^29^, ^30^, ^31^ and has emerged as an imaging technique that renders depth‐resolved microstructural images of biological tissue with endogenous sources of optical contrast.^32^ Previous studies in human brain tissues have demonstrated the visualization of mesoscale features, such as cortical layers, microvasculature networks, and neurons with PS‐OCT.^33^, ^34^, ^35^, ^36^ Complementing PS‐OCT, birefringence microscopy (BRM) allows label‐free, high‐resolution imaging of exclusively myelin‐related changes, because the contrast mechanism of BRM is based uniquely on optical birefringence. Recent improved implementation of BRM specific to imaging myelin integrity at single‐axon resolution^37^ enables quantitative assessment of changes associated with disease.

In summary, both PS‐OCT and BRM systems are sensitive to myelin. We demonstrate the potential for PS‐OCT to image differences in the quantity and density of myelin, and for BRM to image structural changes of myelin at high resolution, offering the potential to provide valuable insights into details of myelin degeneration in association with disease etiology. This approach holds promise for enhancing our understanding of these neurodegenerative conditions by providing practical tools for quantifying myelin structural changes.

## 2 METHODS

### 2.1 Human tissue samples

De‐identified human brain tissues were obtained from 18 cases from the Boston University Alzheimer's Disease Research Center and Understanding Neurologic Injury and Traumatic Encephalopathy (UNITE) brain banks, including five brains with neuropathologically confirmed AD (Braak stages VI) without co‐morbidities, five age‐matched brains with neuropathologically confirmed CTE (McKee stages III–IV) without co‐morbidities, and eight age‐matched normal control (NC) subjects. For this study, four NC samples were excluded due to either the postmortem intervals (PMIs) exceeding 24 h or the presence of brain pathology in the region of interest (ROI). This resulted in a final dataset of five late‐stage AD, five late‐stage CTE, and four NC brains (Table 1). All fixed tissue samples were from the dorsolateral prefrontal cortex (Brodmann areas 9 and 46), with dimensions of 2 cm  × 2 cm × 0.5 cm. Although this is not a brain region that displays early pathological markers, we selected it because it has pronounced accumulation of p‐tau at the late stages of both AD and CTE,^7^, ^8^, ^38^ and because we sought to assess whether local indications of myelin structural degradation precede protein depositions. Of note, PMI did not exceed 24 h for all samples, as longer PMIs are known to yield myelin degradation independent of disease.^39^ All brain samples were fixed in 4% periodate‐lysine‐paraformaldehyde and stored at 4°C for at least 2 months, and then transferred in 10% formalin for a short transportation period. All sample blocks were washed for 1 month in 0.01 M phosphate‐buffered saline (PBS) at 4°C while being shaken gently to remove excess fixation agent in the tissue. After washing, the tissue blocks were embedded in 4.5% agarose and preserved in 0.01 M PBS with 1% sodium azide at 4°C before imaging. The embedded blocks were first imaged by serial‐sectioning (described below) with the PS‐OCT system, and, during the sectioning process, 30‐µm‐thick sections were taken at regular depth intervals for imaging with BRM and for staining. We also performed immunohistochemical staining to determine whether any p‐tau and amyloid plaque changes were consistent with the corresponding diagnosis.

**RESEARCH IN CONTEXT**

1. **Systematic review**: Most studies of myelin integrity in Alzheimer's disease (AD) and chronic traumatic encephalopathy (CTE) focus on white matter, whereas gray matter (GM) myelin changes are less explored. This study uses birefringence microscopy and polarization‐sensitive optical coherence tomography to investigate GM myelin defects in AD and CTE.
2. **Interpretation**: When age and postmortem interval were controlled for, no statistically significant differences in myelin defect density were observed between AD, CTE, and controls, albeit in small cohorts. Age differences between control and disease groups emerged as a critical confounding factor, with previously observed trends becoming non‐significant after age adjustment. Weak correlations between myelin defects and protein pathology suggest myelin changes may occur independent of protein accumulations.
3. **Future directions**: Future studies require larger, age‐matched cohorts to distinguish disease‐specific changes from age‐related alterations. Integrating automated deep‐learning analysis could improve defect quantification accuracy. Studies should examine early disease stages and explore correlations between myelin damage and neuroimaging biomarkers.

**TABLE 1:** Demographic table.

Group	Sample no.	Sex	Age, y	PMI, h	Braak stage	CDS	CDR
Control^a^	1	F	61	4			
	2	M	69	13.5			
	3	M	67	13.5			
	4	M	59	13.92			
CTE	5	M	81	15.75	III	122	
	6	M	89	17.5	III	156	
	7	M	78	7	IV	156	
	8	M	75	16.5	IV	147	
	9	M	86	18.75	III	65	
AD	10	F	84	6.5	VI		3
	11	M	76	3	VI		2
	12	M	86	6.25	VI		2
	13	F	83	10.5	VI		3
	14	M	65	17.75	VI	153	

^a^ Absence NFTs and Aβ pathology in dorsolateral prefrontal cortex, and absence of neurodegenerative disorder diagnosis are main selection criteria.

Abbreviations: Aβ, amyloid beta; AD, Alzheimer's disease; CDR, Clinical Dementia Rating; CDS, Cognitive Difficulties Scale; h, hours; CTE, chronic traumatic encephalopathy; NFTs, neurofibrillary tangle; no., number; PMI, post‐mortem interval; y, years.

The Cognitive Difficulties Scale (CDS) and Clinical Dementia Rating (CDR) scale are used to evaluate cognitive symptoms (such as memory and perception) prior to death, where higher values reflect greater difficulty in performance of cognitive tasks.^40^ Unfortunately, for three of the four NCs, these scores were not available from the brain bank.

### 2.2 Serial‐sectioning PS‐OCT system

A serial sectioning PS‐OCT system^35^ was used to image the samples and slice them into thin sections for further microscopic analysis with BRM. The axial resolution of the PS‐OCT system is ≈6 µm in tissue (with a refractive index of 1.4). A 4× air objective (Olympus, UPLFLN4x, NA 0.13) was in the sample arm, yielding a lateral resolution of ≈5 µm.

The workflow for serial‐sectioning PS‐OCT acquisition has been reported in detail previously.^31^, ^33^, ^41^ During imaging, the brain tissue block was mounted in a bath filled with PBS. The field of view (FOV) for each image tile was 3 mm × 3 mm, and the overlap between adjacent tiles was 15%. A customized vibratome slicer^42^ with a 63.5 mm sapphire blade (DDK, Inc.) was mounted adjacent to the PS‐OCT imaging head to facilitate cutting a slice from the top of the tissue block upon completion of each enface scan of the sample surface. The imaging depth for each PS‐OCT scan (the A‐scan length) is 150 µm, and the corresponding brain tissue was then sliced into five sections of 30‐µm thickness. Those tissue sections were preserved for BRM imaging.

Customized stage and vibratome control software was embedded within the image acquisition software for fully automated serial‐sectioning acquisition. Data transfer and saving were implemented in two sequential steps: (1) from the local acquisition computer to a local storage server after one image tile; and (2) from the storage server to a centralized server at the Boston University Shared Computing Cluster, following the entire serial scan.^43^ A parallelized post‐processing script written in MATLAB was then executed to stitch together the individual image tiles to reconstruct the full volumetric image of the entire sample.

### 2.3 BRM imaging system

BRM imaging was performed sequentially on the slices sectioned and collected after PS‐OCT imaging. Prior to the BRM imaging, the sectioned slices were index‐matched (to reduce scattering) with 85% volume concentration of glycerol with 0.01% sodium azide and then wet‐mounted and cover‐slipped on 1‐mm microscopy slides. Qualitative BRM imaging was performed on 30‐µm sections with a commercial slide‐scanner microscope (Olympus, VS‐120), which was modified with the addition of a narrowband (630 ± 10 nm) filter after the lamp and with left‐ and right‐circular polarizers (API American Polarizers, APNCP37‐105G‐RH‐AR2, and APNCP37‐105G‐AR) in the illumination and detection arms, as shown in Blanke et al.^44^ A 20× air objective (Olympus, 1‐U2B825‐U, NA = 0.75) was used for imaging, which resulted in a lateral resolution of 0.42 µm. Volumetric imaging was accomplished with a 30‐µm z‐stack of 1.3‐µm step size. Each image tile has an FOV of 0.67 mm × 0.67 mm.

### 2.4 Relative retardance estimation with PS‐OCT

The logic for bulk imaging/measurement of relative retardance is that, with myelin degradation, the global structural anisotropy (alignment) is expected to decrease as a result of more random orientations of the optic axis of lipid structures, thereby reducing average birefringence. The relative retardance of the brain tissue was extracted using polarization information from the PS‐OCT images. More specifically, a linear function was fit to estimate the relative retardance, and the slope of the retardance along the depth of an A‐scan was extracted. The retardance accumulation was computed using the amplitudes of the two polarization channels, as described previously in Wang et al.^31^ Linear fitting was performed using a custom script in MATLAB.

After extracting the relative retardance for each image tile, the tiles were stitched with Fiji software, using the coordinates generated during the volumetric reconstruction.

### 2.5 Defining the ROIs

**FIGURE 1:**
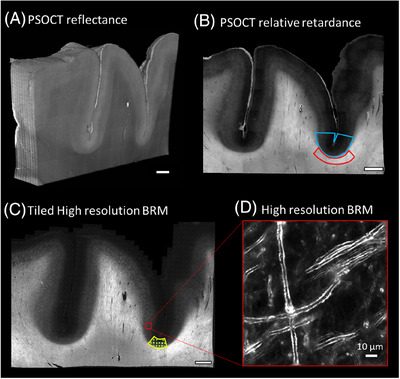
Examples of PS‐OCT and BRM images for the whole sample. (A) PS‐OCT volumetric reflectance image. (B) An example of a relative retardance map extracted from the PS‐OCT signal, where the blue outline traces ROI in GM and red in WM. (C) BRM image of the entire sample with yellow contour outlining the ROI for counting of myelin pathological incidents and green boxes indicating tiles used for annotation; and insert (D) zoomed‐in sample in high resolution. Scale bar: 2 mm. BRM, birefringence microscopy; GM, gray matter; PS‐OCT, polarization‐sensitive optical coherence tomography; ROI, region of interest; WM, white matter.

The sulcus of the dorsolateral prefrontal cortex was manually selected to compare the relative retardance between the pathological and the normal brain samples. One ROI each, for GM and WM, was labeled at each slice for each brain sample. The PS‐OCT relative retardance map was used to identify the boundary between the GM and WM, given the distinct difference between the two. The GM ROI was drawn centered at the bottom of the sulcus with a length of 2 to 3 mm and included the full width of the cortex (blue outline in Figure 1B). The WM ROI was drawn under the GM ROI in the sulcus with a similar length and width (red outline in Figure 1B). These GM and WM ROIs were first manually drawn in Fiji using the polygon selection tool, and then transformed into binary masks from the ROIs using a customized MATLAB script.

GM ROIs were denoted for myelin defects analysis and comparison with p‐tau or Aβ chromogen accumulation. One ≈1 × 1‐mm ROI was manually selected for each sample in layers IV–VI (Figure S1) to investigate the prevalence of pathological myelin defects in BRM images (yellow contour in Figure 1C). Layer IV contains a high content of myelinated axons and therefore shows up as a bright band in both the PS‐OCT retardance map and BRM microscopy images. Layer VI is close to the WM and can be easily distinguished by significant changes in the signal intensity for both PS‐OCT and BRM images.

Using custom software, the 1 × 1‐mm ROI mentioned was divided into multiple smaller images in a randomized grid pattern (see Figure 1C, green squares). Because the complete BRM images captured on the slide scanner are extremely large (250–300 GB), the custom software was developed to manage this data efficiently. The software allows researchers to outline the sulcus ROI and then automatically crop it into manageable 600 × 600 pixel sub‐images (≈195 µm× 195 µm in size) for further annotation. To reduce annotation burden and minimize potential selection bias (e.g., unconscious tendency to select images with more defects for disease cases or fewer defects in NC), the software selects from grid spacing approximately one‐third of all cropped images from each ROI. The software assigns a random name to each sub‐image to ensure blinded analysis. After generating all the images for each case, they were shuffled randomly, placed in a folder, and made available for annotation.

For comparing chromogen accumulation with myelin defect counts, slides were stained for p‐tau and Aβ, and images were manually co‐registered with BRM images using Fiji software. The grid pattern used for myelin defect annotation was then overlaid on the stained images. ROIs were manually drawn to measure chromogen accumulation approximately in the same areas where defects were identified.

### 2.6 Classification of types of pathological myelin defects

**FIGURE 2:**
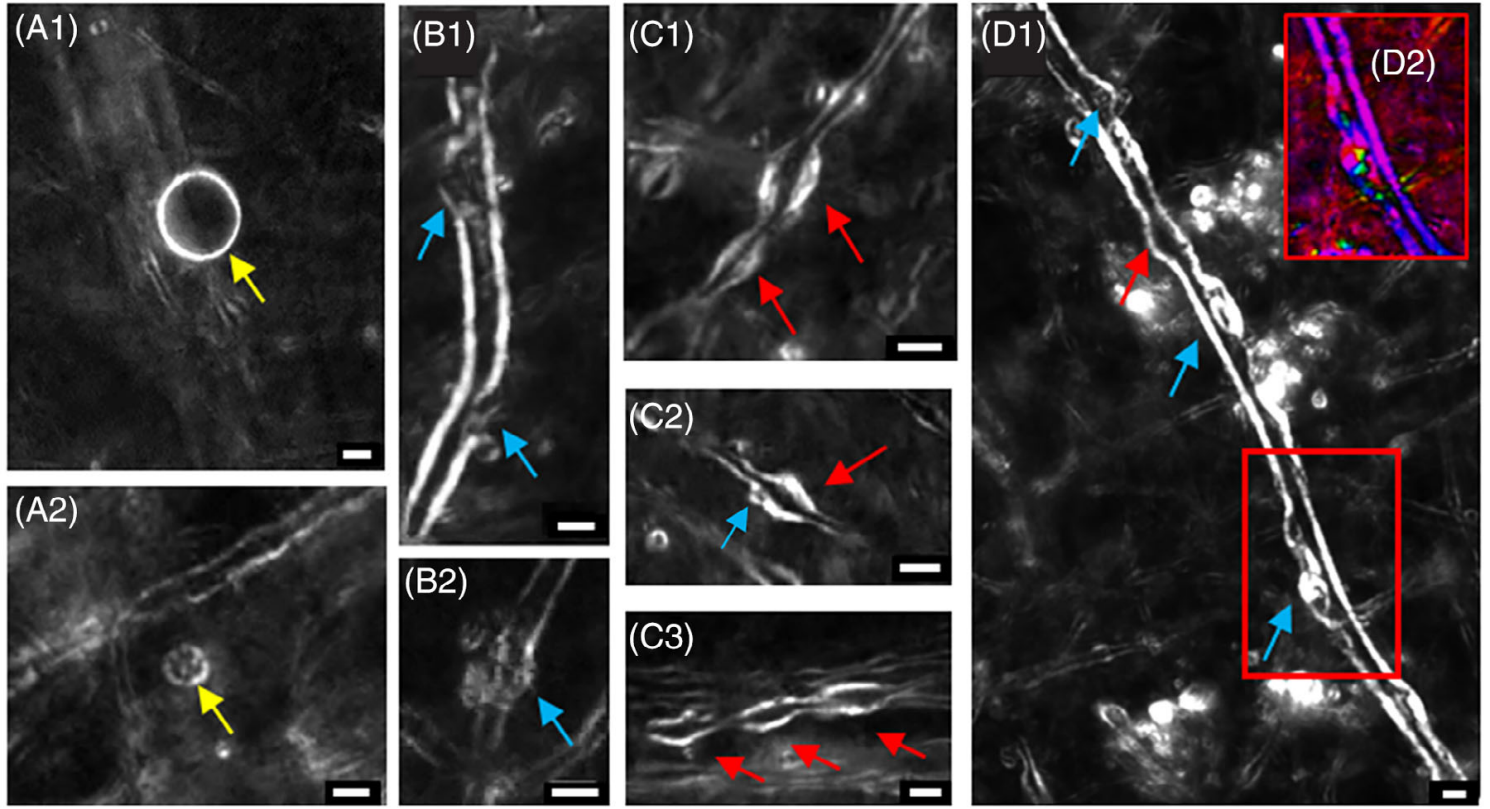
Exemplary myelin defect annotation of high‐resolution BRM images: The yellow arrow in A1 and A2 points to vesicles; the blue arrows in B1, B2, and D1 are delamination/blebbing myelin defects; C1 and C3 are myelin swellings indicated with red arrows; and C2 is an example of a mixed defect where the myelin swelling is larger. D2 shows an orientation in qBRM image of the myelin defect. Scale bar: 5 µm. BRM, birefringence microscopy; qBRM, quantitative birefringence microscopy.

Structural changes associated with myelin pathologies were readily visualized with high‐resolution BRM images of individual myelinated axons (Figures 1D and 2). At high resolution, the annotator was able to identify individual myelin pathological features (structural defects) as small as 1 µm. During the annotation, the images were loaded randomly to avoid bias. In the course of the study, we identified different types of myelin defects. Future investigations with larger numbers of tissue samples will address the question of whether different classes of defects are associated with different stages or types of disease.

In BRM analysis, “myelin defect” is defined as any observable structural aberration from normal, healthy myelin sheath. Normal myelin in BRM images, for fibers that are approximately parallel to the tissue section plane, appears as two parallel running lines with consistent diameter along their length, exhibiting continuous, smooth, and uniform birefringence. Myelin defects, by contrast, represent any deviation from this normal appearance, including localized abrupt changes in diameter, disruptions in the continuity of the myelin sheath, delaminations, or formation of detached myelin‐derived structures. These defects were identified through careful examination of the three‐dimensional (3D) BRM z‐stack data, allowing us to distinguish genuine structural abnormalities from imaging artifacts.

Myelin defects were categorized into three classes based on their imaged morphological features: myelin swellings, myelin delamination/blebbing, and isolated lipid membrane vesicles (assumed to be derived from detached myelin lipids). Myelin swellings are defined as a myelin pathological abnormality, where the myelin sheath is intact but visually appears as bulging or swelling from the inside (Figure 2C1–C3). Based on reported electron microscopy (EM) studies, the possible content within the bulging could be the dense cytoplasm that is granular and contains vacuoles and lysosomes.^45^ The delaminations and blebbings are defects that impact the integrity of the myelin sheath by splitting and ballooning/fragmenting of the myelin sheath (Figure 2B1,B2,D1).^45^ Under some circumstances, the defects appear as a combination of the above‐mentioned abnormalities and were annotated as either myelin swellings or delamination/blebbing, depending on which defect was more prominent (Figure 2C2, blue arrow).

The isolated vesicles are spherical structures with birefringent properties appearing as small bright rings visible in only one to two planes of z‐stack images (Figure 2A1,A2). Our identification of these structures as vesicles derived from myelin debris is based on several key factors. From a physical perspective, birefringence in brain tissue originates predominantly from highly ordered, multilayered lipid structures, with myelin being the primary source of this optical property. The strong birefringent signal observed in these vesicular structures indicates that they contain organized lipid layers similar to those found in myelin sheaths.

Similar vesicular structures have been observed in other studies of myelin pathology using the same BRM technique. Notably, Blanke et al.^44^ employed an identical BRM methodology and reported the formation of comparable myelin‐derived vesicles following cortical injury in a rhesus macaque model, where such structures were absent in uninjured control tissue. In addition, EM studies by Peters and Folger^45^ have documented that myelin degradation often involves the formation of detached spherical vesicles composed of myelin lipids, which are consistent with the structures we observe in our BRM images. Based on these findings, we conclude that these isolated vesicular structures represent myelin debris formed during the process of myelin degradation.

### 2.7 Defect annotation process

To ensure robust identification of myelin defects, three independent annotators examined the entire dataset. We implemented a consensus approach where only defects identified by at least two annotators were considered true positives. For this purpose, we employed an algorithm requiring a minimum Intersection over Union (IoU) of 20% between annotations to be considered the same structure, with a tolerance of ±1 *z*‐plane to account for minor differences in annotators' perception of optimal focus. When annotators disagreed on myelin defect classification (e.g., one identified a structure as delamination, whereas another classified it as swelling), these defects were categorized as “mixed” to acknowledge the morphological complexity that prevented unanimous classification.

### 2.8 Protocol for immunohistochemical staining

Following BRM imaging, we used the same tissue sections for p‐tau and amyloid plaque staining for improved correlation analysis of myelin structural changes. (This is possible because BRM is a label‐free method.)

Thionine staining (for nucleic acids) was applied to 30‐µm‐thick sections as described previously.^46^ The stained slides were imaged with a commercial slide‐scanner (Olympus VS120) and a 20x objective. This was performed as follows: The slide coverslips were removed and the mounted 30‐µm‐thick sections were placed in acetone at −20°C for 30 min. Slides were then rinsed in water for 5 min and placed in PBS. Slides were then placed on the automated slide preparation system Discovery Ultra (Ventana), which was run on the recommended protocols for the Chromomap 3,3'‐diaminobenzidine (DAB) dye kit (Roche Diagnostics). Briefly, heat‐induced epitope retrieval and background blocking steps were followed by primary antibody incubation with either AT8 (1:100, Invitrogen MN1020) or Ab4G8 (1:15000, BioLegend 800701) for 1 h, followed by horseradish peroxidase (HRP)‐DAB chromogenic reaction. Sections were counterstained with hematoxylin.

When staining protocols were completed, the slides were placed in water, dehydrated in xylene on the Leica Autostainer XL system, and then hand cover‐slipped using a Leica Surgipath Micromount. The slides were imaged using the Zeis AxioScan.Z1 slide scanner.

### 2.9 Aβ and p‐tau stain analysis

Polygonal ROIs were drawn around the group of ROIs as seen in Figure 1C, and the selected areas were scaled for approximately the same GM sulcus region on the stained 30‐µm sections. The ImageJ^47^ color‐threshold function was used to separate and select DAB‐positive regions from hematoxylin. The selected threshold could not differentiate neuromelanin and DAB; however, the neuromelanin component was considered negligible. Areas occupied by DAB as positive for p‐tau or Aβ were proportioned to the area of the polygonal ROI.

### 2.10 Statistical analysis

For each sample, we calculated the average number of myelin defects per mm^2^ based on 9–27 images (mean: 16 per sample), depending on sulcus morphology. To analyze differences in myelin defect prevalence among groups, we employed a linear mixed‐effects (LME) model. In addition, we used the Bonferroni correction for multiple comparisons between groups. The number of myelin defects per unit area served as the dependent variable, group membership (NC, CTE, AD) as the fixed effect, and PMI and age as covariates to control for potential confounding effects. Individual patient identification (ID) was included as a random effect to account for within‐subject correlations across multiple measurements.

Similarly, for relative retardance measured with PS‐OCT, an LME model was employed with relative retardance as the response variable, group membership as the fixed effect, PMI and age as covariates, and patient ID as a random effect. Measurements were averaged across ROIs from each 150‐µm slice, with 10–22 measurement points per sample (mean: 14 per sample).

To examine relationships between myelin defects and protein pathology, linear regression analysis was conducted with the number of myelin defects as the dependent variable and AT8/Ab4G8 chromogen percent area as the independent variable, controlling for PMI. Standardized coefficients (β) were reported to indicate the direction (positive or negative) and strength of correlations, along with corresponding significance levels.

All statistical analyses were performed using IBM SPSS Statistics (version 27). Statistical significance was defined as *p* < 0.05, with Bonferroni corrections for multiple comparisons.

## 3 RESULTS

### 3.1 PS‐OCT measurement of relative retardance

**FIGURE 3:**
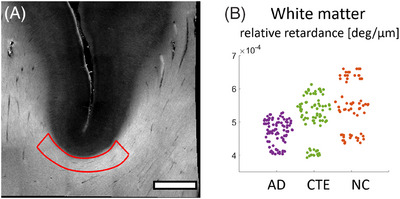
(A) Exemplary relative retardance map where red ROI outlines WM. The swarm chart of relative retardance for each slice in the respective ROI in white matter (B). Scale bar: 2 mm. AD, Alzheimer's disease; CTE, chronic traumatic encephalopathy; NC, normal control; ROI, region of interest; WM, white matter.

All 14 brain tissue samples were volumetrically reconstructed with our PS‐OCT postprocessing pipeline, as detailed in Yang et al. (2022).^33^ Each sample yielded 10–22 slices, each 150‐µm thick, for tissue blocks with total block thickness of 1.5–3.3 mm. Each slice was imaged by stitching together 100 tiles (10 × 10 tiles: Figure 2A). To assess the impact of age as a confounding factor, we performed LME analyses using two covariate models: (1) PMI only and (2) PMI and age together. GM results were excluded for PS‐OCT images due to poor signal‐to‐noise ratios resulting from substantially fewer myelinated axons compared to WM. In WM, when using PMI as the sole covariate, relative retardance demonstrated a nearly significant negative trend in AD (*p* = 0.021), whereas CTE exhibited a non‐significant negative trend (*p* = 0.980). However, when both PMI and age were included as covariates, the AD effect became non‐significant (*p* = 0.093), demonstrating that age differences between groups substantially influenced the initial findings (Figure 3; Tables S1 and S2A,B). The comparison between single and dual covariate models illustrates the critical importance of controlling for age in neurodegenerative disease research.

### 3.2 BRM quantification of myelin defects

**FIGURE 4:**
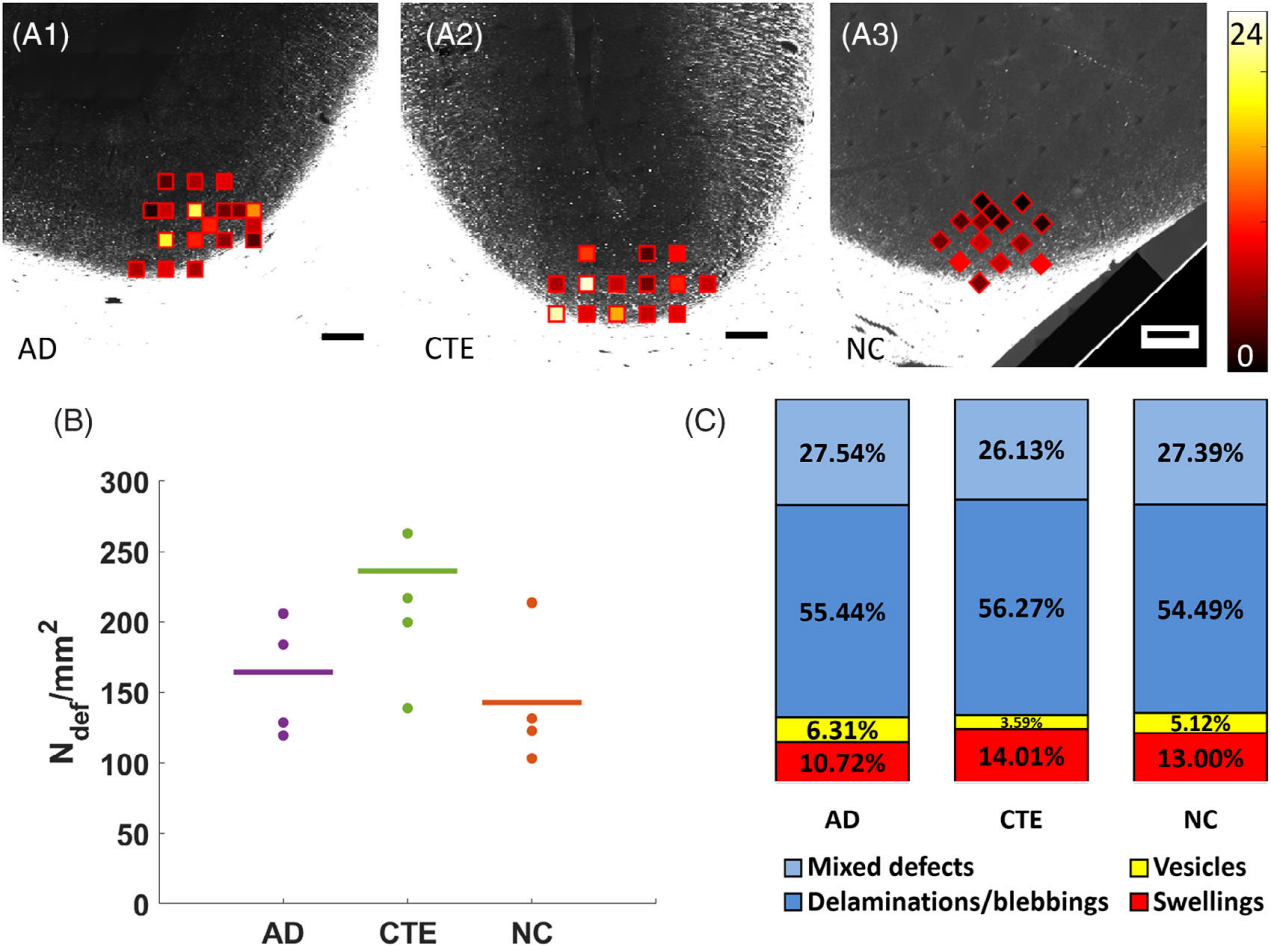
(A1–A3) Total number of defects found in each ROI color‐coded with a heatmap, where black represents zero defects and white is the maximum number of defects found in all ROIs. (B) Scatter plot of the average number of myelin abnormalities per mm^2^ in each sample, where the line indicates each group's mean value. (C) Stacked bar graph representing each group's defect type ratio. Scale bar: 250 µm. AD, Alzheimer's disease; CTE, chronic traumatic encephalopathy; N_def_/mm^2^, number of defects per mm^2^; NC, normal control; ROI, region of interest.

Due to the dense myelin packing in the WM, annotation of the myelin defects with BRM was performed only for the GM ROIs (Figure 4A). The number of defects per unit area found in each group is shown in Figure 4B, where mean values are reported in Table S1. Similar to the PS‐OCT analysis, we compared LME models with PMI only vs PMI and age as covariates. When using PMI alone, the CTE group showed a nearly significant increase in myelin defects compared to NC (*p* = 0.070). However, when age was added as a covariate, this effect became non‐significant (*p* = 0.298), with the overall group effect changing from *p* = 0.162 to *p* = 0.370 (Table S3A,B). Neither PMI nor age, individually, showed significant effects on myelin defect density in the dual covariate model (*p* = 0.848 and *p* = 0.861, respectively). These findings demonstrate that age differences between groups rather than disease‐specific processes were driving the previously observed trends.

Microscopic analysis of myelin defects revealed a predominance of delamination/blebbing alterations (Figure 4C). The second largest category comprised “mixed” defects, where the morphological complexity prevented annotators from reaching unanimous classification on structural subtype. These mixed defects often displayed characteristics of multiple alteration types, highlighting the complex nature of myelin pathology in neurodegenerative conditions.

### 3.3 Additional observations

**FIGURE 5:**
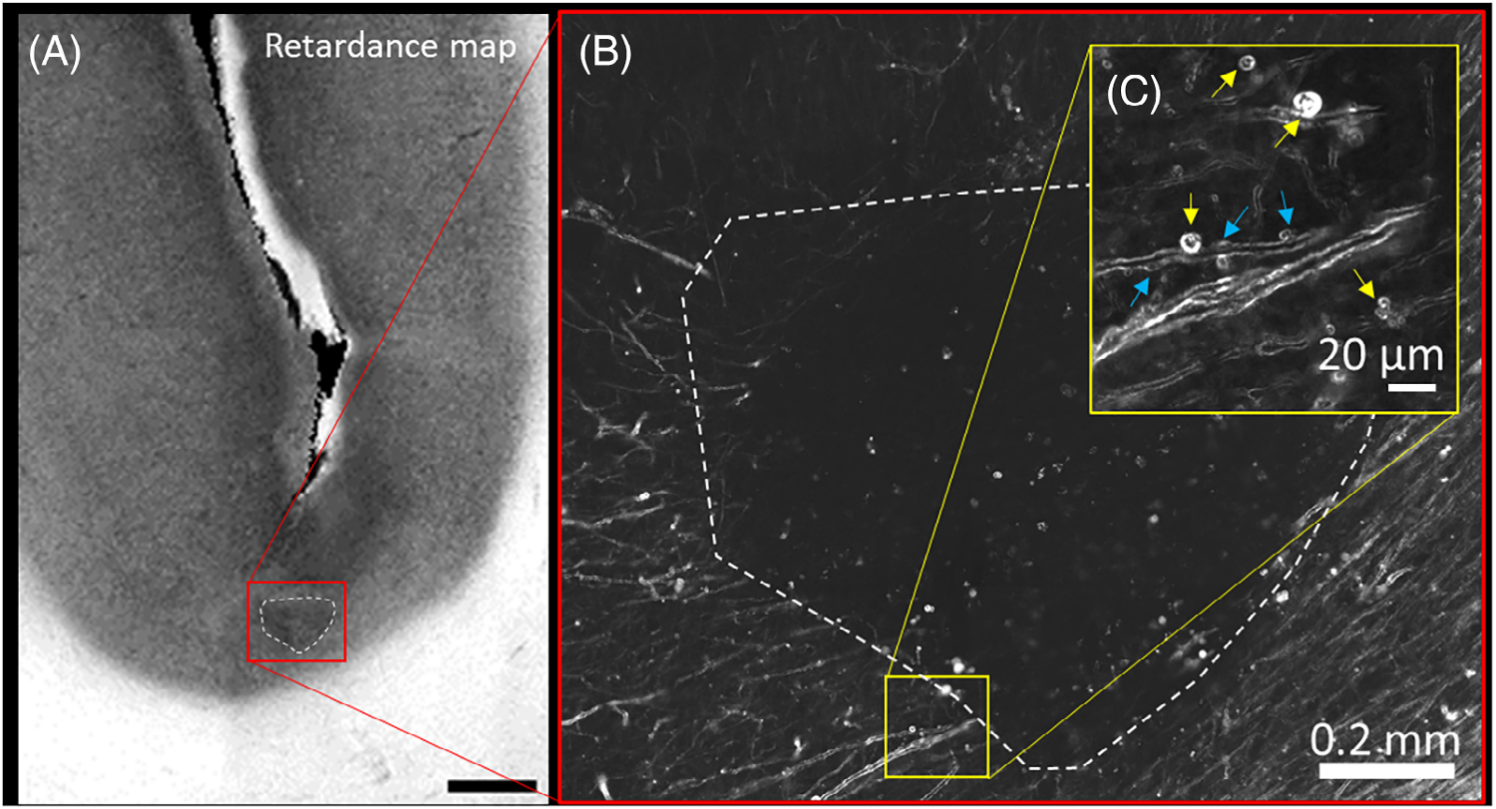
(A) Relative retardance map for the CTE sample with a glial scar. (B) High‐resolution BRM of the glial scar with a void of myelin in the center. (C) Myelin defects (yellow arrows–vesicles, blue arrows–delamination/blebbing) in the area surrounding the scar. A white dashed line outlines the glial scar (A, B). Scale bar: 1 mm. BRM, birefringence microscopy; CTE, chronic traumatic encephalopathy.

The presence of a glial scar was identified in one of the CTE samples in the sulcus region (Figure 5). Relative retardance in the area of the glial scar has lower values compared to areas less affected by the scar (Figure 5A). High‐resolution BRM images reveal the absence of myelin inside the glial scar (Figure 5B) and an increase in different types of myelin defects in areas surrounding the scar (Figure 5C).

As expected, the value of close to 0% for AT8 staining in NC is consistent with other studies that confirm that less p‐tau deposition is correlated with healthy subjects.^48^ NC images presented little or no staining with Ab4G8, and the clear separation of the GM and WM layers was observed. For both AT8 and Ab4G8, staining melanin granules found in neuron cell bodies (Figure S2) sometimes caused a small amount of brown color measurement in NC samples because of the color thresholding method, but this effect was considered negligible.

**FIGURE 6:**
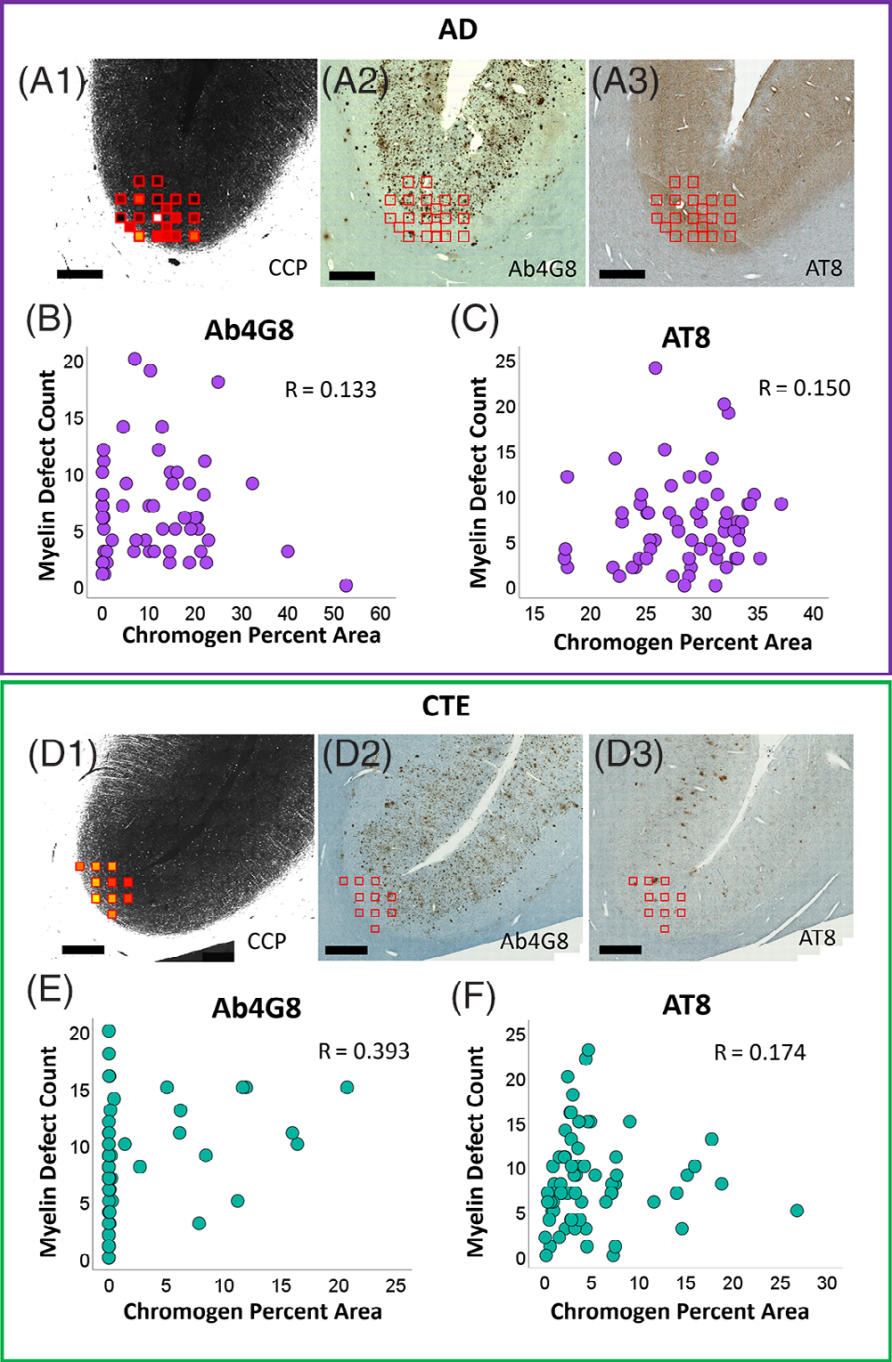
(A1, D1) The individual ROIs chosen from the sulcus region of a representative AD and CTE case, respectively, on a BRM image. (A2, D2) Immunohistochemistry staining of Ab4G8 shows the Aβ distribution in the same section as BRM. (A3, D3) Immunohistochemistry staining of AT8, showing the distribution of p‐tau in the same case, on a tandem section. Scatter plots for the number of myelin defects to percent occupied by Ab4G8 staining (B, E), where each dot represents one ROI of Ab4G8 stained AD and CTE cases, and R reports the Spearman correlation coefficient. (C, F) Similar scatter plots but for AT8 staining. Scale bar: 1 mm. Aβ, amyloid beta; AD, Alzheimer's disease; BRM, birefringence microscopy; CTE, chronic traumatic encephalopathy; p‐tau, phosphorylated tau; ROI, region of interest.

We used a scatter plot to visualize any potential correlation between the myelin defect count and the chromogen (AT8 and Ab4G8) positive percent area. In Figure 6, each dot represents one ROI per case across disease groups. For AD cases, Spearman's correlation analysis showed a weak positive correlation between myelin defect count and AT8 (*R* = 0.150, *p* = 0.243; Table S4 and Figure 6C) and Ab4G8 staining (*R* = 0.133, *p* = 0.308; Table S5 and Figure 6B). In CTE cases, we found a significant positive correlation between defect counts and Ab4G8 (*R* = 0.393, *p* = 0.003; Table S6 and Figure 6E) but only a weak, non‐significant correlation with AT8 staining (*R* = 0.174, *p* = 0.155; Table S7 and Figure 6F). However, when controlling for PMI in linear regression models, none of these correlations remained significant. In the CTE for the Ab4G8 model, PMI emerged as a significant predictor (*β* = 0.558, *p* < 0.001), whereas chromogen percent area did not (*β* = 0.064, *p* = 0.615). Similarly, the relationships between myelin defects and protein pathology markers were not significant in the regression models of AD for AT8 (*β* = 0.105, *p* = 0.421), Ab4G8 (*β* = 0.074, *p* = 0.575), and in CTE for AT8 (*β* = 0.005, *p* = 0.967).

## 4 DISCUSSION

As stated, although most of the literature on myelin changes in AD and CTE focus on WM, we decided to focus on GM, as this is where most of the observed early neuropathological changes occur.^8^, ^24^ We examined the myelin sheaths of individual axons and the myelin pathological defects in GM using high‐resolution BRM images. Our annotation and characterization of myelin defects in BRM images are consistent with myelin defects identified in the EM literature.^45^ We characterized the severity of the myelin pathology in each brain sample by quantifying the number of defects per unit area, providing insight into the myelin pathology associated with these diseases. When controlling for age differences among groups, our analysis revealed no statistically significant differences in myelin defect density between AD, CTE, and NC groups, suggesting that age‐related myelin changes may overpower disease‐specific findings in studies with inadequate age matching and with limited cohort numbers.

We offer the following hypothesis to explain the difference in myelin defect prevalence between AD and CTE. In AD, oligodendrocyte damage caused by cholesterol metabolism dysregulation^22^, ^49^ leads to an overall reduction in myelination. In addition, compared with CTE, there are fewer axons left in AD (indicated by retardance decrease in AD), and there is less myelin debris due to neurodegeneration‐related cell death.^50^ In CTE, however, oligodendrocytes and neurons are more focally damaged by trauma rather than resulting from an ongoing metabolic disturbance, causing the emergence of irregular myelinated structures immediately from the impact or after myelin repair,^51^ which, we submit, explains the higher counts of myelin defects.

### 4.1 Methodological considerations and confounding factors

Our counting of myelin defects relies on manual annotation, which may limit accuracy. However, there are no automated methods to evaluate myelin degradation in BRM images due to the challenges of 3D data annotation and the novelty of this imaging modality. Of note, to avoid unintentional bias caused by manual annotation, we blinded the annotators to the sample being annotated, and the samples were randomly sequenced. The productivity of our pipeline can be further enhanced by incorporating deep‐learning–based methods, such as convolutional neural networks,^52^, ^53^ to segment these defects automatically and further quantify the pathology on a larger scale. Such development is ongoing and will be incorporated in future publications.

Although our findings demonstrate myelin structural changes in AD and CTE, we acknowledge that neuroinflammatory processes may play a role in these changes. All cases were evaluated for active inflammation, and no acute neuroinflammatory conditions were found. The cases were not analyzed quantitatively for microglial activation, a common feature of CTE. Beyond the remote and well‐healed glial scar in one case, we observed no microhemorrhages or infarcts with our label‐free imaging. However, our methodology was not optimized for detecting neuroinflammatory markers. We suggest that future studies correlating PS‐OCT and BRM findings with immunohistochemical analyses of microglial activation (Iba1, CD68) and astrocytic reactivity (glial fibrillary acidic protein) would provide insights into the relationship between myelin degradation and inflammation in these conditions.

Although we cannot rule out the contribution of a sub‐diagnostic disease process, the cases were comprehensively analyzed neuropathologically for co‐morbid conditions, and no identifiable inflammatory or neurodegenerative conditions were found. Neurodegenerative conditions rarely present in isolation, with mixed pathologies increasingly common in aging populations.^54^ Various pathological states could potentially contribute to myelin damage, including inflammatory disorders, metabolic disorders, and vascular conditions that compromise blood supply to WM regions.^54^ WM integrity can be affected by multiple pathophysiological processes simultaneously, with abnormalities potentially emerging before clinical symptom manifestation.^55^ The trend toward higher myelin defect prevalence in our CTE samples could reflect differences in subtle pathological mechanisms rather than disease‐specific mechanisms alone, as our results suggest that age‐related confounders may significantly influence myelin pathology measurements.

Several parameters are known to affect myelin integrity in the human brain, including age, which is associated with cognitive decline in healthy elderly individuals.^56^, ^57^ As such, the age difference between our NC group (64 ± 4.7 years, mean ± SD) and disease groups (80.3 ± 7 years) represents a significant confounding factor, as age‐related myelin breakdown follows developmental patterns, with late‐myelinating regions being more vulnerable. When we included age as a covariate, previously observed trends became non‐significant, emphasizing the need for closer age‐matching or larger sample sizes in future studies. Another parameter of concern is the PMI, the time between death and fixing/preserving of the brain tissue. PMI‐related changes in myelin integrity (potentially due to autolysis) have been identified in both EM and magnetic resonance imaging (MRI) studies.^39^, ^58^ Glausier et al. found a positive trend in the number of myelin defects with longer PMI; however, this effect was statistically insignificant within the first 24 h.^59^ Consequently, we selected brain samples with PMI less than 24 h for this study. Environmental temperature between death and fixation also affects tissue preservation, particularly lipid‐rich structures like myelin.^60^ Although standard protocols were likely employed for all samples, specific temperature data during the post‐mortem interval is unavailable, remaining a potential uncontrolled variable.

### 4.2 Role of myelin degradation in the etiology of AD and CTE

Myelin is required for normal brain function, as it insulates axons and enables fast action‐potential propagation.^61^ Myelin damage not only decreases transmission speed but also significantly affects the refractory period of axons, which in turn can lead to deterioration in cognitive function.^22^ The link between myelination and neurodegeneration is reinforced by Braak's discovery that the progression of NFTs in AD counters the normal temporal pattern of myelination during neurodevelopment.^38^ Vulnerability theories suggest that thinner myelin sheaths and metabolic alteration of oligodendrocytes formed in later stages of neurodevelopment are more susceptible to stressors such as iron, cholesterol, hypoxia, and oxidative stress.^22^

Another proposition implicates toxicity of NFTs and Aβ in oligodendrocyte/myelin damage.^62^ In one of the proposed mechanisms, damaged oligodendrocytes release more iron, which can serve as a core of forming Aβ, and in a positive‐feedback loop, further induces stronger toxicity.^22^ It is important to note that studies of early AD stages propose theories that myelin degradation precedes amyloid/tau.^63^

Oligodendrocyte cholesterol accumulation due to apolipoprotein E (*APOE*) ε4 (the allele associated with AD) affects myelination, axons, and cognition.^49^ Alternatively, the amyloid hypothesis posits myelin changes stem from Aβ/NFT‐induced neuron/synapse loss.^64^ Oligodendrocyte demise might result from an Aβ‐triggered nSMase‐ceramide cascade.^65^ *As amyloid‐clearing therapies face challenges*,^66^ *alternate AD pathogenesis theories gain more interest*.^67^, ^68^

### 4.3 Multi‐modal imaging insights and implications for future research

*We submit that the optical methods reported here offer the opportunity to assess the development of myelin degradation in earlier stages of AD, importantly before significant development of Aβ/NFT*.

Our optical methodologies complement emerging MRI techniques for myelin assessment. Although ultra‐high‐field ex vivo MRI can image whole brain samples at 100–200 µm resolution,^69^ our PS‐OCT (5–6 µm) and BRM (0.42 µm) approaches enable visualization of single‐axon abnormalities undetectable at MRI resolutions. PS‐OCT serves as an ideal bridging technology because its serial sectioning preserves 3D tissue architecture with minimal distortion, facilitating direct registration with MRI data.^31^ This multi‐scale approach combines MRI's whole‐brain coverage with our optical methods' detailed microstructural information, providing mechanistic insights that cannot currently be achieved through MRI alone. Although myelin degradation research in CTE is limited, evidence supporting our findings^70^, ^71^, ^72^ aligns with decreased myelin/oligodendrocyte markers. Notably, CTE and AD exhibit differing affected transcripts: iron metabolism/stress response in CTE and myelination/maturation/axonal guidance in AD.^19^ Mouse studies strengthen the pre‐tau myelin damage theory.^51^

Despite the literature suggesting an association between myelin damage and disease‐hallmark pathological protein accumulations, in our samples we observed only weak, if any, correlations between myelin defects and Aβ or p‐tau for the brain region studied, underscoring the complexity of the relationship between these entities in neurodegeneration. Although we evaluated only late disease stages, and a larger study is needed to decipher whether myelin damage is an early event or just a result of the ongoing changes during neurodegeneration, *we submit that our unlabeled* *optical imaging methods are valuable in studying myelin structure, as lipid structure is preserved by our methods, and large volumes of tissue can be imaged*.

In summary, we imaged 14 human brain samples to explore the differences associated with advanced AD and CTE (compared to NC). Our methodology proved to be easy to implement and scalable to larger throughputs. To statistically and rigorously assess the relation between myelin degradation and neurodegenerative diseases, future work will require larger, carefully age‐matched cohorts to address current limitations and will also aim to study the relationships between myelin defect incidence and MRI‐derived parameters, while incorporating deep‐learning methods to facilitate high‐throughput quantification of myelin defects.

In conclusion, we report novel and scalable imaging methods that offer the potential to deepen our understanding of myelin integrity changes in AD and CTE, and how they relate to disease etiology. These insights can contribute to a broader understanding of neurodegenerative diseases and potentially help guide diagnostic or therapeutic strategies.

## CONFLICT OF INTEREST STATEMENT

All authors report no conflict of interest in relation to this study. Any author disclosures are available in the Supporting Information.

## CONSENT STATEMENT

The brain tissue samples used in this study were obtained from the Boston University Alzheimer's Disease Research Center (BU ADRC) and the UNITE Brain Bank. All samples were collected post‐mortem, fully de‐identified, and provided under established institutional protocols. According to institutional and federal guidelines, the use of de‐identified post‐mortem human tissues does not constitute human subjects research and, therefore, does not require informed consent. This study was conducted in compliance with the ethical standards of the BU ADRC, UNITE Brain Bank, and applicable regulations.

## Supporting information



Supporting Information

Supporting Information

Supporting Information

Supporting Information

Supporting Information

Supporting Information

Supporting Information

Supporting Information

Supporting Information

Supporting Information
