# Supplementary material for: Imaging myelin degradation in ex vivo prefrontal cortex tissue blocks in Alzheimer's disease and chronic traumatic encephalopathy
Source: Alzheimers Dement. 2025 Aug 22;21(8):e70582. doi: 10.1002/alz.70582 (PMC12371461; doi:10.1002/alz.70582)
Supplement: Supplementary file 1 — Supporting Information [file ALZ-21-e70582-s009.pdf]

Supplementary Table 1. Mean and standard deviation values of relative retardance and the number of defects across groups

Parameter	AD	CTE	NC
Relative retardance in WM, [deg/μm]	$(4.72 \pm 0.45) \times 10^{-4}$	$(5.15 \pm 0.66) \times 10^{-4}$	$(5.47 \pm 0.68) \times 10^{-4}$
Number of defects per mm^2	164±38	236±83	142±48
