# Supplementary material for: Imaging myelin degradation in ex vivo prefrontal cortex tissue blocks in Alzheimer's disease and chronic traumatic encephalopathy
Source: Alzheimers Dement. 2025 Aug 22;21(8):e70582. doi: 10.1002/alz.70582 (PMC12371461; doi:10.1002/alz.70582)
Supplement: Supplementary file 2 — Supporting Information [file ALZ-21-e70582-s005.pdf]

Supplementary Table 2A. Comparison between WM relative retardance in different groups using LME model with PMI as a covariate.

Type III Tests of Fixed Effects[a]

Source	Numerator df	Denominator df	F	Sig.
Intercept	1	16.699	233.433	.000
Category	2	16.684	4.052	.037
PMI	1	16.689	4.672	.045

a. Dependent Variable: retardance.

Estimates of Fixed Effects[a]

Parameter	Estimate	Std. Error	df	t	Sig.	98% Confidence Interval	
						Lower Bound	Upper Bound
Intercept	.000622	4.325524E-5	16.675	14.371	.000	.000510	.000733
[Category=AD]	-9.126810E-5	3.566697E-5	16.662	-2.559	.021	-.000183	4.775010E-7
[Category=CTE]	-9.230271E-7	3.687444E-5	16.703	-.025	.980	-9.575041E-5	9.390436E-5
[Category=NC]	0[b]	0	.	.	.	.	.
PMI	-6.655123E-6	3.078998E-6	16.689	-2.161	.045	-1.457384E-5	1.263599E-6

a. Dependent Variable: retardance.

b. This parameter is set to zero because it is redundant.

Supplementary Table 2B. Comparison between WM relative retardance in different groups using LME model with PMI and age as covariates.

Type III Tests of Fixed Effects[a]

Source	Numerator df	Denominator df	F	Sig.
Intercept	1	15.657	9.193	.008
Category	2	15.671	3.397	.060
PMI	1	15.679	4.200	.058
age	1	15.647	.000	.988

a. Dependent Variable: retardance.

Estimates of Fixed Effects[a]

Parameter	Estimate	Std. Error	df	t	Sig.	98% Confidence Interval	
						Lower Bound	Upper Bound
Intercept	.000619	.000169	15.653	3.667	.002	.000182	.001056
[Category=AD]	-9.179617E-5	5.139304E-5	15.656	-1.786	.093	-.000225	4.129732E-5
[Category=CTE]	-1.603844E-6	5.967544E-5	15.687	-.027	.979	-.000156	.000153
[Category=NC]	0[b]	0	.	.	.	.	.
PMI	-6.648220E-6	3.243957E-6	15.679	-2.049	.058	-1.504777E-5	1.751328E-6
age	3.664866E-8	2.465060E-6	15.647	.015	.988	-6.347570E-6	6.420868E-6

a. Dependent Variable: retardance.

b. This parameter is set to zero because it is redundant.
