# Supplementary material for: Imaging myelin degradation in ex vivo prefrontal cortex tissue blocks in Alzheimer's disease and chronic traumatic encephalopathy
Source: Alzheimers Dement. 2025 Aug 22;21(8):e70582. doi: 10.1002/alz.70582 (PMC12371461; doi:10.1002/alz.70582)
Supplement: Supplementary file 3 — Supporting Information [file ALZ-21-e70582-s004.pdf]

Supplementary Table 3A. Comparison of GM myelin defect density in different groups using the LME model with PMI as a covariate.

Type III Tests of Fixed Effects[a]

Source	Numerator df	Denominator df	F	Sig.
Intercept	1	9.346	17.093	.002
Category	2	9.733	2.208	.162
PMI	1	9.700	.060	.811

a. Dependent Variable: defects.

Estimates of Fixed Effects[a]

Parameter	Estimate	Std. Error	df	t	Sig.	98% Confidence Interval	
						Lower Bound	Upper Bound
Intercept	5.910432	1.963694	9.087	3.010	.015	.381038	11.439826
[Category=AD]	.680826	1.633843	9.423	.417	.686	-3.886256	5.247909
[Category=CTE]	3.486975	1.722947	10.247	2.024	.070	-1.253744	8.227694
[Category=NC]	0[b]	0	.	.	.	.	.
PMI	-.034861	.141840	9.700	-.246	.811	-.429122	.359400

a. Dependent Variable: defects.

b. This parameter is set to zero because it is redundant.

Supplementary Table 3B. Comparison of GM myelin defect density in different groups using the LME model with PMI and age as covariates.

Type III Tests of Fixed Effects[a]

Source	Numerator df	Denominator df	F	Sig.
Intercept	1	8.968	.392	.547
Category	2	9.011	1.111	.370
PMI	1	8.767	.039	.848
age	1	9.002	.032	.861

a. Dependent Variable: defects.

Estimates of Fixed Effects[a]

Parameter	Estimate	Std. Error	df	t	Sig.	98% Confidence Interval	
						Lower Bound	Upper Bound
Intercept	4.517989	7.865024	8.808	.574	.580	-17.773525	26.809504
[Category=AD]	.381321	2.433050	9.402	.157	.879	-6.422790	7.185431
[Category=CTE]	3.111746	2.824547	9.402	1.102	.298	-4.787130	11.010622
[Category=NC]	0[b]	0	.	.	.	.	.
PMI	-.029798	.150868	8.767	-.198	.848	-.457832	.398237
age	.020798	.115437	9.002	.180	.861	-.304887	.346482

a. Dependent Variable: defects.

b. This parameter is set to zero because it is redundant.
