# Supplementary material for: Imaging myelin degradation in ex vivo prefrontal cortex tissue blocks in Alzheimer's disease and chronic traumatic encephalopathy
Source: Alzheimers Dement. 2025 Aug 22;21(8):e70582. doi: 10.1002/alz.70582 (PMC12371461; doi:10.1002/alz.70582)
Supplement: Supplementary file 4 — Supporting Information [file ALZ-21-e70582-s008.pdf]

Supplementary Table 4. Correlation and linear regression analysis between myelin defect count and AT8 chromogen percent area in AD cases, controlling for post-mortem interval (PMI).

Correlations[a]

			Myelin Defect Count	Chromogen Percent Area
Spearman's rho	Myelin Defect Count	Correlation Coefficient	1.000	.150
		Sig. (2-tailed)	.	.243
		N	81	62
	Chromogen Percent Area	Correlation Coefficient	.150	1.000
		Sig. (2-tailed)	.243	.
		N	62	62

a. Disease Groups = AD

Coefficients[a,b]

Model		Unstandardized Coefficients B	Unstandardized Coefficients Std. Error	Standardized Coefficients Beta	t	Sig.
1	(Constant)	2.883	4.116		.700	.486
	Chromogen Percent Area	.110	.135	.105	.811	.421
	PMI	.086	.125	.089	.689	.493

a. Disease Groups = AD

b. Dependent Variable: Myelin Defect Count
