# Supplementary material for: Imaging myelin degradation in ex vivo prefrontal cortex tissue blocks in Alzheimer's disease and chronic traumatic encephalopathy
Source: Alzheimers Dement. 2025 Aug 22;21(8):e70582. doi: 10.1002/alz.70582 (PMC12371461; doi:10.1002/alz.70582)
Supplement: Supplementary file 6 — Supporting Information [file ALZ-21-e70582-s007.pdf]

Supplementary Table 6. Correlation and linear regression analysis between myelin defect count and Ab4G8 chromogen percent area in CTE cases, controlling for post-mortem interval (PMI).

Correlations[a]

			Myelin Defect Count	Chromogen Percent Area
Spearman's rho	Myelin Defect Count	Correlation Coefficient	1.000	.393**
		Sig. (2-tailed)	.	.003
		N	68	54
	Chromogen Percent Area	Correlation Coefficient	.393**	1.000
		Sig. (2-tailed)	.003	.
		N	54	54

**. Correlation is significant at the 0.01 level (2-tailed).

a. Disease Groups = CTE

Coefficients[a,b]

Model		Unstandardized Coefficients B	Unstandardized Coefficients Std. Error	Standardized Coefficients Beta	t	Sig.
1	(Constant)	-33.373	9.253		-3.607	.001
	Chromogen Percent Area	.063	.123	.064	.507	.615
	PMI	2.440	.552	.558	4.421	.000

a. Disease Groups = CTE

b. Dependent Variable: Myelin Defect Count
