# Supplementary material for: Imaging myelin degradation in ex vivo prefrontal cortex tissue blocks in Alzheimer's disease and chronic traumatic encephalopathy
Source: Alzheimers Dement. 2025 Aug 22;21(8):e70582. doi: 10.1002/alz.70582 (PMC12371461; doi:10.1002/alz.70582)
Supplement: Supplementary file 7 — Supporting Information [file ALZ-21-e70582-s001.pdf]

Supplementary Table 7. Correlation and linear regression analysis between myelin defect count and AT8 chromogen percent area in CTE cases, controlling for post-mortem interval (PMI).

Correlations[a]

			Myelin Defect Count	Chromogen Percent Area
Spearman's rho	Myelin Defect Count	Correlation Coefficient	1.000	.174
		Sig. (2-tailed)	.	.155
		N	68	68
	Chromogen Percent Area	Correlation Coefficient	.174	1.000
		Sig. (2-tailed)	.155	.
		N	68	68

a. Disease Groups = CTE

Coefficients[a,b]

Model		Unstandardized Coefficients B	Unstandardized Coefficients Std. Error	Standardized Coefficients Beta	t	Sig.
1	(Constant)	8.979	2.429		3.697	.000
	Chromogen Percent Area	.005	.125	.005	.041	.967
	PMI	-.031	.152	-.025	-.203	.840

a. Disease Groups = CTE

b. Dependent Variable: Myelin Defect Count
