# Supplementary material for: Imaging myelin degradation in ex vivo prefrontal cortex tissue blocks in Alzheimer's disease and chronic traumatic encephalopathy
Source: Alzheimers Dement. 2025 Aug 22;21(8):e70582. doi: 10.1002/alz.70582 (PMC12371461; doi:10.1002/alz.70582)
Supplement: Supplementary file 8 — Supporting Information [file ALZ-21-e70582-s002.pdf]

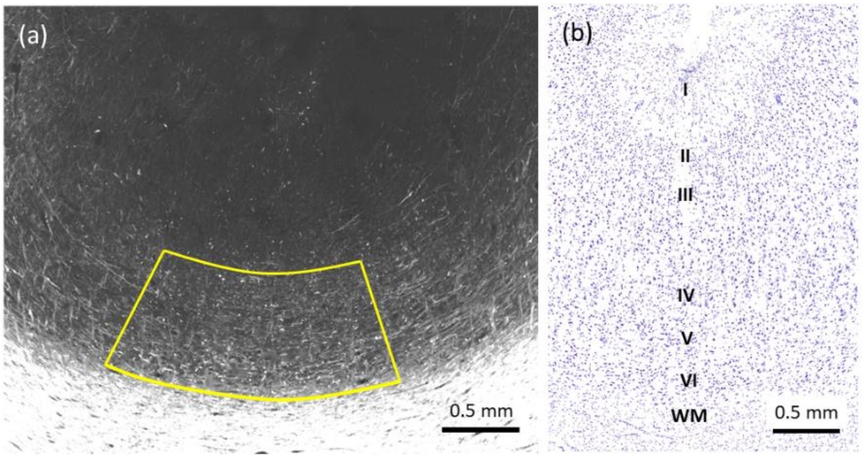

Supplementary Figure 1. (a) high-resolution BRM image will yellow contoured ROI for image analysis; (b) thionine staining of the sample to confirm the cortical layers.
