# Supplementary material for: Imaging myelin degradation in ex vivo prefrontal cortex tissue blocks in Alzheimer's disease and chronic traumatic encephalopathy
Source: Alzheimers Dement. 2025 Aug 22;21(8):e70582. doi: 10.1002/alz.70582 (PMC12371461; doi:10.1002/alz.70582)
Supplement: Supplementary file 9 — Supporting Information [file ALZ-21-e70582-s006.pdf]

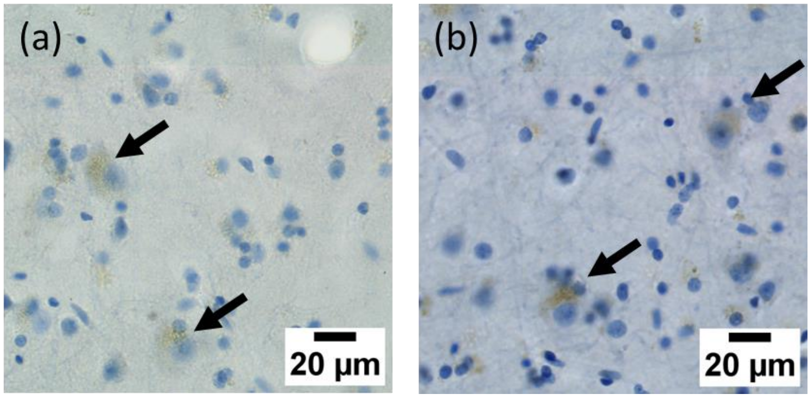

Supplementary Figure 2. Melanin granules (pointed with black arrows) found in neuron cell bodies of NC samples in (**a**) AT8 and (**b**) Ab4G8 stained sections.
